# Impact of antiplatelet therapy on erectile dysfunction following percutaneous coronary intervention in patients with ST-segment elevation myocardial infarction

**DOI:** 10.3389/fmed.2026.1874442

**Published:** 2026-09-03

**Authors:** Joongwon Choi, Chung Un Lee, Hyun Woong Park, Jung Hoon Kim, Yong Seong Lee, Jong-Hwa Ahn, Jin Sin Koh, Min Gyu Kang, Weon Kim, Udaya S. Tantry, Paul A. Gurbel, Jin-Yong Hwang, Young-Hoon Jeong

**Affiliations:** 1 Department of Urology, Chung-Ang University Gwangmyeong Hospital, Gwangmyeong and Chung-Ang University College of Medicine, Seoul, Republic of Korea; 2 Division of Cardiology, Department of Internal Medicine, Chungnam National University Hospital, Chungnam National University School of Medicine, Daejeon, Republic of Korea; 3 Division of Cardiology, Gyeongsang National University Changwon Hospital, Changwon, Republic of Korea; 4 Department of Internal Medicine, Gyeongsang National University School of Medicine, Jinju, Republic of Korea; 5 Division of Cardiology, Gyeongsang National University Hospital, Jinju, Republic of Korea; 6 Cardiovascular Department of Internal Medicine, Kyung Hee University Hospital, Seoul, Republic of Korea; 7 Sinai Center for Thrombosis Research and Drug Development, Sinai Hospital of Baltimore, Baltimore, MD, United States; 8 CAU Thrombosis and Biomarker Center, Chung-Ang University Gwangmyeong Hospital, Gwangmyeong, Republic of Korea; 9 Department of Internal Medicine, Chung-Ang University College of Medicine, Seoul, Republic of Korea

**Keywords:** antiplatelet, bleeding, erectile dysfunction, myocardial infarction, P2Y12 inhibitor

## Abstract

**Background:**

Acute myocardial infarction (AMI) is strongly associated with erectile dysfunction (ED). This study evaluated the association between P2Y_12_ receptor inhibitor type and ED, and factors associated with moderate-to-severe ED in high-risk patients with AMI.

**Methods:**

The HEALING-AMI trial randomized patients with naïve ST-segment elevation myocardial infarction (STEMI) to dual antiplatelet therapy (DAPT) with either ticagrelor or clopidogrel. This substudy included 59 male patients who completed 6 months of DAPT (ticagrelor, *n* = 30; clopidogrel, *n* = 29). Erectile function [International Index of Erectile Function-5 (IIEF-5)], Bleeding Academic Research Consortium (BARC) bleeding, and dyspnoea were assessed using standardized questionnaires.

**Results:**

At 6-month follow-up, moderate-to-severe ED (IIEF-5 ≤ 11) and bleeding episodes (predominantly BARC type 1) were observed in 47.5% and 54.2%, respectively. Compared with clopidogrel, ticagrelor was associated with a lower IIEF-5 score (9.7 ± 8.8 vs. 17.0 ± 7.8, *p* = 0.002) and lower platelet reactivity [32 ± 67 vs. 146 ± 55 P2Y12 reaction units (PRU) measured by VerifyNow, *p* < 0.001]. Moderate-to-severe ED was more common in patients treated with ticagrelor than in those treated with clopidogrel (63.3% vs. 31.0%). Bleeding episodes and platelet reactivity were also significantly associated with moderate-to-severe ED. In multivariable analysis, both P2Y_12_ inhibitor type and bleeding episodes were independently associated with moderate-to-severe ED. After multivariable adjustment, ticagrelor-treated patients who experienced bleeding had an approximately 5.6-fold higher odds of moderate-to-severe ED than clopidogrel-treated patients without bleeding (95% CI 1.052–30.087, *p* = 0.044).

**Conclusions:**

In male patients with STEMI, moderate-to-severe ED and bleeding episodes were common during DAPT. P2Y_12_ inhibitor type (ticagrelor vs. clopidogrel) and bleeding episodes were associated with moderate-to-severe ED.

## 1 Introduction

Acute myocardial infarction (AMI) is a life-threatening cardiovascular (CV) event resulting from abrupt coronary artery occlusion that restricts blood flow to the myocardium. AMI is also closely linked to erectile dysfunction (ED), as the two conditions share a common vascular pathophysiology. In high-risk patients with AMI, early intervention and guideline-directed medical therapy (GDMT) have improved clinical outcomes. Dual antiplatelet therapy (DAPT), consisting of aspirin plus a P2Y_12_ inhibitor, is recommended as standard care after percutaneous coronary intervention (PCI) in patients with AMI (1). Beyond its contribution to coronary occlusion, platelet accumulation within the injured myocardium amplifies local inflammation and promotes left ventricular remodeling (2, 3).

Oral adenosine diphosphate (ADP) receptor P2Y_12_ inhibitors include thienopyridines (ticlopidine, clopidogrel, prasugrel) and ticagrelor. Thienopyridines are prodrugs that irreversibly inhibit P2Y_12_ via short-lived active metabolites, whereas ticagrelor—an adenosine triphosphate (ATP) analog—directly and reversibly binds P2Y_12_ as a noncompetitive allosteric antagonist (4). Compared with clopidogrel, ticagrelor provides more potent platelet inhibition. Ticagrelor also exerts pleiotropic effects beyond platelet P2Y_12_ inhibition that may explain its mortality benefit. These effects are most commonly adenosine-mediated and may modulate vascular tone and microcirculation and influence inflammation and reperfusion biology (5).

Contemporary data on the prevalence of ED and its associated factors in this population remain limited, and the impact of different antiplatelet regimens on ED is not well defined. We therefore evaluated the prevalence and factors associated with ED in patients with AMI treated with contemporary procedural strategies and optimal medical therapy.

## 2 Patients and methods

### 2.1 . Design and patients

The High Platelet Inhibition with Ticagrelor to Improve Left Ventricular Remodeling in Patients with ST-Segment Elevation Myocardial Infarction (HEALING-AMI) study (NCT02224534) was a prospective, randomized, multicentre trial conducted at 10 academic centers in South Korea (3). Patients were eligible for enrolment if they experienced a first ST-segment elevation myocardial infarction (STEMI) treated with primary PCI and were assigned to a DAPT regimen.

**Figure 1:**
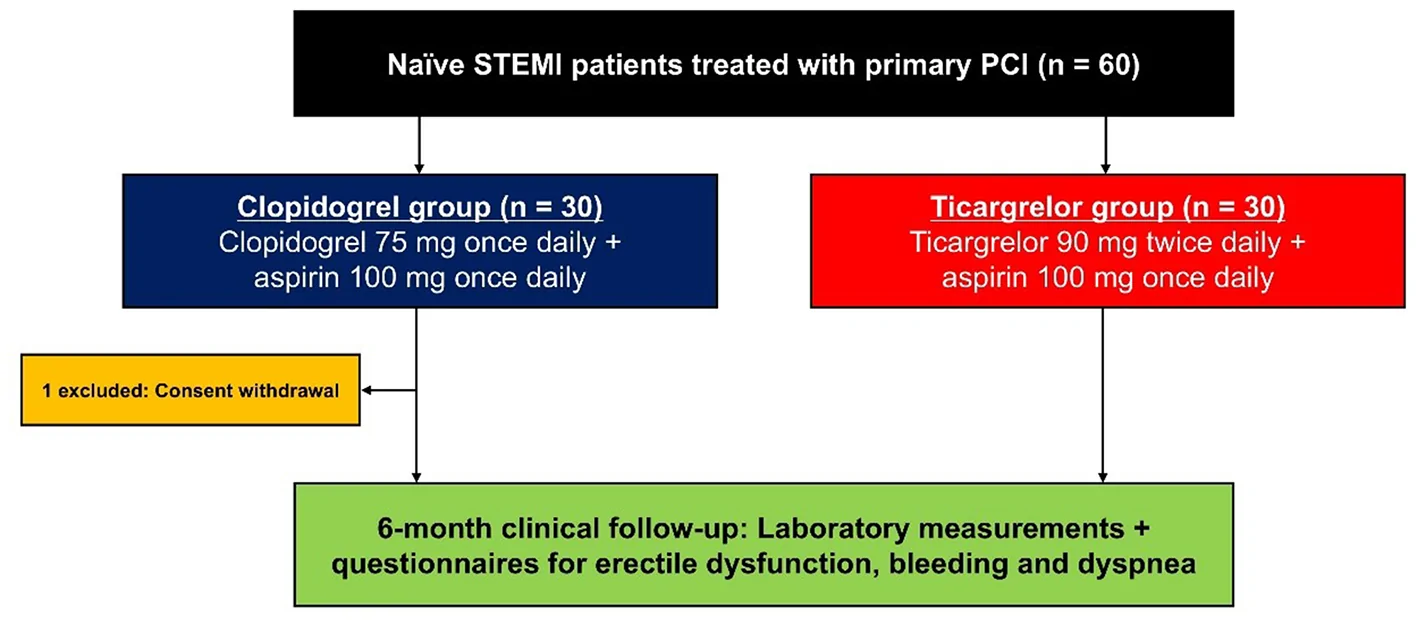
Flow diagram of the study. PCI, percutaneous coronary intervention; STEMI, ST-segment elevation myocardial infarction.

The HEALING-AMI–ED substudy evaluated the prevalence and factors associated with ED during medical therapy after primary PCI in male patients with STEMI. This substudy was conducted exclusively at Gyeongsang National University Hospital in Jinju, South Korea (Figure 1). The study protocol was separately approved by the Institutional Review Board of each participating institute (IRB No. 4-2013-0764), and all patients provided written informed consent.

### 2.2 . Randomization and procedures

Patients who met all clinical criteria and provided written informed consent were randomly assigned to receive a 180 mg ticagrelor or 600 mg clopidogrel loading dose (1:1) in the emergency room before the index PCI. All patients received 300 mg of aspirin orally.

All PCI procedures were performed according to standard techniques (6). After PCI, patients who received ticagrelor continued ticagrelor 90 mg twice daily, whereas those who received clopidogrel continued clopidogrel 75 mg once daily throughout the study period. Both groups continued to take 100 mg of aspirin daily indefinitely, and all patients were recommended to receive GDMT.

During the 6-month follow-up, data regarding clinical status and adverse events were collected at 1, 3, and 6 months. Adherence to pharmacological therapy was also assessed through meticulous interviews, tablet counting, and dedicated questionnaires.

### 2.3 . Questionnaire and definitions

After completion of 6-month DAPT, the research coordinators collected data using a dedicated questionnaire including ED grade, bleeding episodes, and dyspnoea (Supplementary Table 1). ED was evaluated using the International Index of Erectile Function-5 (IIEF-5) score. Scores range from 5 to 25; higher scores indicate better function. Scores of 22–25 indicate no ED, 17–21 mild ED, 12–16 mild-to-moderate ED, 8–11 moderate ED, and 5–7 severe ED (7). Because patients presented emergently with STEMI and underwent immediate primary PCI, administering the IIEF-5 questionnaire before treatment initiation was not feasible. Erectile function was therefore assessed only at the 6-month follow-up, and the present study focused on erectile function at that time point rather than longitudinal changes from baseline.

The Bleeding Academic Research Consortium (BARC) criteria were used to identify bleeding episodes (8, 9). Dyspnoea was categorized using the modified Medical Research Council (mMRC) scale (10).

### 2.4 . Laboratory measurements

Baseline biochemical assessments were performed using blood samples drawn from an antecubital vein after arrival at the emergency department and at the 6-month follow-up visit in the clinic. Platelet function testing was performed using the VerifyNow PRUTest (Werfen, Barcelona, Spain), with the optical signal reported as P2Y12 reaction units (PRU). Baseline samples were collected before coronary angiography, and follow-up platelet function testing was performed at 1 month, at least 2 hours after DAPT administration. Platelet reactivity was assessed at three prespecified time points—before loading, at hospital discharge, and at the 1-month follow-up—using the VerifyNow P2Y12 assay. The 1-month value was prespecified as the index measure of maintenance-phase platelet reactivity because the preloading value preceded P2Y_12_ inhibitor exposure, whereas the discharge value could have been influenced by loading-dose effects and the acute phase of myocardial infarction. No additional platelet function testing was performed during subsequent follow-up.

### 2.5 . Vascular measurements

Vascular function was measured on admission and at the 6-month post-PCI follow-up visit. Using an automated Doppler ultrasound device (VP-1,000; Colin Co., Ltd., Komaki, Japan), the ankle-brachial index (ABI) was measured in each leg within 48 h after PCI. To evaluate arterial stiffness (media function), brachial-ankle pulse wave velocity (baPWV) was measured using the same noninvasive device. Pulse wave velocity was calculated as PWV = ΔD/ΔT, where ΔD is the distance traveled, and ΔT is the transit time. Endothelial function was assessed at the 6 mo follow-up using the EndoPAT system (version 3.0.4; Itamar Medical, Caesarea, Israel) in consenting patients (11).

### 2.6 . Endpoints

The primary endpoint was the presence of moderate-to-severe erectile dysfunction (IIEF-5 ≤ 11) at the 6-month follow-up. Secondary endpoints were bleeding episodes and dyspnoea. We also evaluated the factors associated with moderate-to-severe ED. Moderate-to-severe ED was defined as an IIEF-5 score of ≤ 11, corresponding to moderate or severe ED (7).

### 2.7 . Statistical analysis

Continuous variables were reported as mean ± standard deviation or as median (interquartile range [IQR]). The Kolmogorov–Smirnov test was used to assess the normality of continuous variables. Continuous variables were compared using Student's *t*-test when normally distributed. Categorical variables were reported as numbers and percentages and compared using the chi-square test or Fisher's exact test. Receiver operating characteristic (ROC) curve analysis was performed to determine optimal cutoffs for continuous variables. The ROC curve analysis was performed as an exploratory analysis to identify a potential PRU threshold for subsequent hypothesis-generating analyses. The discriminative performance of PRU was assessed using the area under the curve (AUC) analysis. To evaluate factors associated with moderate-to-severe ED at the 6-month follow-up, variables with *p* < 0.1 in univariate analysis were entered into multivariable analysis to estimate odds ratios (ORs) and 95% confidence intervals (CIs). Given the limited sample size and number of outcome events, we intentionally restricted the number of variables included in the final models to minimize the risk of overfitting. With 28 outcome events, model 1 estimated three regression coefficients, yielding an events-per-variable (EPV) ratio of 9.3, whereas model 2 estimated four regression coefficients, yielding an EPV of 7.0. A *p* < 0.05 was considered statistically significant. Statistical analyses were performed using SPSS Statistics for Windows, version 27.0 (IBM Corp., Armonk, NY, USA).

## 3 Results

Initially, 60 male patients with STEMI were enrolled. After one patient in the clopidogrel group withdrew consent, 30 participants in the ticagrelor group and 29 in the clopidogrel group completed the questionnaires and laboratory measurements at the 6-month follow-up (Figure 1).

### 3.1 . P2Y_12_ inhibitor type: baseline characteristics, laboratory measurements, and questionnaires

Baseline characteristics did not differ significantly between the groups (Table 1). Baseline laboratory measurements also did not differ between the groups (Table 2). After 6-month treatment, the ticagrelor group had a lower mean glomerular filtration rate (GFR) (83 ± 13 vs. 99 ± 27 ml/min/1.73 m^2^; *p* = 0.005) and low-density lipoprotein cholesterol (59 ± 12 vs. 74 ± 21 mg/dl; *p* = 0.001) than the clopidogrel group. As expected, ticagrelor provided a more potent antiplatelet effect than clopidogrel (32 ± 67 vs. 146 ± 55 PRU; *p* < 0.001).

**Table 1:** Baseline characteristics according to type of P2Y_12_ inhibitor.

Characteristics	Clopidogrel group	Ticagrelor group	*p* value
	(***n*** = **29)**	**(*****n*** = **30)**	
Age, year old	52.3 ± 7.9	53.5 ± 6.6	0.509
Male, *n* (%)	29 (100)	30 (100)	1.000
BMI, kg/m^2^	25.7 ± 3.3	25.3 ± 3.3	0.690
Killip class, *n* (%)			0.638
I	20 (69.0)	19 (63.3)	
II	6 (20.7)	7 (23.3)	
14.6-7.2,-1.3499ptIII	3 (10.3)	4 (13.3)	
Risk factors or past history, *n* (%)			
Diabetes mellitus	4 (13.8)	2 (6.7)	0.635
Hypertension	11 (37.9)	8 (26.7)	0.518
Dyslipidemia	16 (55.2)	13 (43.3)	0.516
Current smoking	21 (72.4)	21 (70.0)	0.838
Chronic kidney disease	2 (6.9)	1 (3.3)	0.976
Peripheral artery disease	0 (0)	1 (3.3)	1.000
Previous stroke	0 (0)	1 (3.3)	1.000
COPD	1 (3.4)	0 (0.0)	0.986
Alcohol consumption			0.920
Non-drinker	15 (51.7)	17 (56.7)	
Moderate drinker	12 (41.4)	12 (40.0)	
15.6-7.2,-1.3499ptHeavy drinker	2 (6.9)	1 (3.3)	
Discharge medication, *n* (%)			
Aspirin	29 (100)	30 (100)	1.000
Statin			1.000
Rosuvastatin 20 mg a day	29 (100)	30 (100)	
Beta blocker			0.617
Carvedilol	21 (72.4)	22 (73.3)	
Nebivolol	5 (17.2)	7 (23.3)	
Angiotensin blockade	25 (86.2)	28 (93.3)	0.635
Calcium channel blocker	1 (3.4)	0 (0.0)	0.986
Proton pump inhibitor	25 (86.2)	26 (86.7)	1.000
Loop diuretic	1 (3.4)	2 (6.7)	1.000
Spironolactone	1 (3.4)	0 (0.0)	0.492
Nitrate	4 (13.8)	0 (0.0)	0.112
Trimetazidine	2 (6.9)	0 (0.0)	0.457
Procedural data			
Pre-PCI TIMI flow grade, *n* (%)			0.672
0	23 (79.3)	22 (73.3)	
1	1 (3.4)	2 (6.7)	
2	5 (17.2)	6 (20.0)	
Post-PCI TIMI flow grade, *n* (%)			1.000
2	2 (6.9)	2 (6.7)	
3	27 (93.1)	28 (93.3)	
	**(*****n*** = **29)**	**(*****n*** = **30)**	
Multivessel disease, *n* (%)	13 (44.8)	16 (53.3)	0.514
Multivessel PCI, *n* (%)	3 (10.3)	3 (10.0)	0.965
Infarct-related artery, *n* (%)			0.072
Left anterior descending	20 (69.0)	19 (63.3)	
Left circumflex	6 (20.7)	7 (23.3)	
Right coronary	3 (10.3)	4 (13.3)	
Intervention method, *n* (%)			0.112
Drug-eluting stent	26 (89.7)	30 (100)	
Bioresorbable scaffold	3 (10.3)	0 (0.0)	
Number of stents	1.1 ± 0.3	1.1 ± 0.3	0.400

BMI, body mass index; COPD, chronic obstructive pulmonary disease; PCI, percutaneous coronary intervention; TIMI, Thrombolysis in Myocardial Infarction.

**Table 2:** Laboratory measurements according to type of P2Y_12_ inhibitor.

Laboratory measurements	Clopidogrel group	Ticagrelor group	*p* value
	(***n*** = **29)**	**(*****n*** = **30)**	
Baseline lab data: on-admission			
WBC, x10^3^/mm^3^	12.3 ± 3.8	11.0 ± 2.6	0.138
Hb, g/dL	14.9 ± 1.8	15.0 ± 1.5	0.763
Platelet, x10^3^/mm^3^	262.8 ± 63.4	255.4 ± 67.5	0.665
GFR, ml/min/1.73 m^2^ (MDRD)	88 ± 18	85 ± 17	0.474
Total cholesterol, mg/dL	203.7 ± 49.2	194.5 ± 52.4	0.487
Triglyceride, mg/dL	224.3 ± 147.0	230.1 ± 183.0	0.893
HDL-C, mg/dL	42.3 ± 12.9	39.6 ± 10.2	0.380
LDL-C, mg/dL	141.0 ± 46.7	133.1 ± 40.0	0.487
VerifyNow, PRU	242 ± 44	241 ± 55	0.898
NT-pro-BNP, pg/mL	617 ± 461	760 ± 746	0.402
LV ejection fraction, %	53.0 ± 6.9	53.7 ± 6.2	0.684
Pulse wave velocity, m/sec	1.48 ± 0.23	1.43 ± 0.24	0.441
Ankle-brachial index	1.1 ± 0.1	1.1 ± 0.1	0.973
Follow-up lab data: 6 months			
WBC, x10^3^/mm^3^	8.2 ± 2.8	7.5 ± 2.0	0.328
Hb, g/dL	14.5 ± 1.4	14.5 ± 1.4	0.988
Platelet, x10^3^/mm^3^	242.3 ± 61.6	241.1 ± 58.5	0.939
GFR, ml/min/1.73 m^2^ (MDRD)	99 ± 27	83 ± 13	0.005
Total cholesterol, mg/dL	135.0 ± 22.1	117.6 ± 18.0	0.002
Triglyceride, mg/dL	170.3 ± 97.0	151.0 ± 88.7	0.427
HDL-C, mg/dL	44.8 ± 13.5	43.4 ± 12.1	0.677
LDL-C, mg/dL	74.3 ± 20.8	58.7 ± 11.9	0.001
VerifyNow, PRU^a^	146 ± 55	32 ± 67	< 0.001
NT-pro-BNP, pg/ml	246 ± 449	137 ± 140	0.217
LV ejection fraction, %	55.3 ± 7.2	56.1 ± 5.8	0.635
Pulse wave velocity, m/sec	1.37 ± 0.18	1.36 ± 0.18	0.614
Ankle-brachial index	1.1 ± 0.1	1.1 ± 0.1	0.690
EndoPAT	(*n* = 18)	(*n* = 18)	
RHI, %	1.7 ± 0.4	1.9 ± 0.6	0.442

a VerifyNow assay was evaluated at 1-month follow-up, whereas other lab measures were conducted at 6-month follow-up.

Hb, hemoglobin; HDL-C, high-density lipoprotein cholesterol; GFR, glomerular filtration rate; LDL-C, low-density lipoprotein cholesterol; LV, left ventricular; MDRD, Modification of Diet in Renal Disease; NT-pro-BNP, N-terminal-pro-Brain Natriuretic Peptide; PRU, P2Y12 reaction units; RHI, reactive hyperemia index; WBC, white blood cell.

In the 6-month questionnaires (Table 3), patients receiving ticagrelor had significantly lower IIEF-5 scores than those receiving clopidogrel (9.7 ± 8.8 vs. 17.0 ± 7.8; *p* = 0.002). The ticagrelor group also had significantly lower scores across all five domains (all *p* values ≤ 0.015). At the 6-month follow-up, moderate-to-severe ED was more common among patients treated with ticagrelor than among those treated with clopidogrel (63.3% vs. 31.0%; *p* = 0.013). Bleeding events were predominantly minor: among the 32 patients who experienced at least one bleeding episode, 31 (96.9%) had BARC type 1 bleeding only, one (3.1%) had BARC type 2 bleeding, and none experienced BARC type 3–5 bleeding (Table 3).

**Table 3:** Questionnaires at 6 months: erectile dysfunction, bleeding and dyspnea according to type of P2Y_12_ inhibitor.

Questionnaire item	Clopidogrel group	Ticagrelor group	*p* value
	(***n*** = **29)**	**(*****n*** = **30)**	
ED category by IIEF-5, n(%)			0.028
No (22–25)	9 (31.0)	2 (6.7)	
Mild (17–21)	10 (34.5)	8 (26.7)	
Mild to moderate (12–16)	1 (3.4)	1 (3.3)	
Moderate (8–11)	6 (20.7)	6 (20.0)	
Severe (5–7)	3 (10.3)	13 (43.3)	
Moderate-to-severe ED, *n* (%)	9 (31.0)	19 (63.3)	0.013
IIEF-5 score	17.0 ± 7.8	9.7 ± 8.8	0.002
Erectile function	3.4 ± 1.7	2.0 ± 1.9	0.004
Orgasmic function	3.4 ± 1.5	2.3 ± 1.8	0.015
Sexual desire	3.5 ± 1.6	1.9 ± 1.9	0.001
Intercourse satisfaction	3.3 ± 1.6	1.8 ± 1.9	0.001
Overall satisfaction	3.3 ± 1.6	1.7 ± 1.8	0.001
Bleeding (BARC), *n* (%)	12 (41.4)	20 (66.7)	0.051
Type 1	12 (41.4)	19 (63.3)	
Type 2	0 (0.0)	1 (3.3)	
Type 3	0 (0.0)	0 (0.0)	
Type 5	0 (0.0)	0 (0.0)	
Dyspnea (mMRC), *n* (%)			0.851
Grade 0	21 (72.4)	22 (73.3)	
Grade 1	8 (27.6)	7 (23.3)	
Grade 2	0 (0.0)	1 (3.3)	
Grade 3	0 (0.0)	0 (0.0)	
Grade 4	0 (0.0)	0 (0.0)	

BARC, Bleeding Academic Research Consortium; ED, erectile dysfunction; IIEF-5, International Index of Erectile Function-5; mMRC, modified Medical Research Council.

Bleeding events were more frequent in the ticagrelor vs. clopidogrel group, predominantly BARC type 1 (66.7% vs. 41.4%; *p* = 0.051). In contrast, dyspnoea incidence did not differ significantly between the groups (26.7% vs. 27.6%; *p* = 0.851).

### 3.2 . ED classification: baseline characteristics, laboratory measurements, and questionnaires

Baseline characteristics, including comorbidities and concomitant medications, were similar across ED categories, except for P2Y_12_ inhibitor type (Table 4). Patients with moderate-to-severe ED were more likely to receive ticagrelor than those with no-to-mild ED (67.9% vs. 35.5%; *p* = 0.026). Laboratory measurements on admission and at follow-up did not differ between the groups, except for the 1-month VerifyNow results (Table 5). Moderate-to-severe ED was significantly associated with a more potent antiplatelet effect (59.7 ± 76.8 vs. 113.2 ± 82.5 PRU; *p* = 0.013). In the 6-month questionnaires (Table 6), moderate-to-severe ED was significantly associated with bleeding episodes (71.5% vs. 41.5%; *p* = 0.046), whereas no association was observed with dyspnea (35.7% vs. 19.4%; *p* = 0.121).

**Table 4:** Baseline characteristics according to ED classification.

Characteristics	No-to-mild ED (*n* = 31)	Moderate-to-severe ED (*n* = 28)	*p* value
Age, year old	53.4 ± 6.7	52.4 ± 7.8	0.627
Male, *n* (%)	31 (100)	28 (100)	1.000
BMI, kg/m^2^	25.6 ± 3.8	25.4 ± 2.7	0.755
Killip class, *n* (%)			0.341
I	18 (58.1)	21 (75.0)	
II	9 (29.0)	4 (14.3)	
III	4 (12.9)	3 (10.7)	
Risk factors or past history, *n* (%)			
Diabetes mellitus	4 (12.9)	2 (7.1)	0.764
Hypertension	10 (32.3)	9 (32.1)	1.000
Dyslipidemia	16 (51.6)	13 (46.4)	0.891
Current smoking	23 (74.2)	19 (67.9)	0.804
Chronic kidney disease	2 (6.5)	1 (3.6)	1.000
Peripheral artery disease	0 (0.0)	1 (3.6)	0.959
Previous stroke	1 (3.2)	0 (0.0)	1.000
COPD	0 (0.0)	1 (3.6)	0.959
Alcohol consumption			0.161
Non-drinker	14 (45.2)	18 (64.3)	
Moderate drinker	14 (45.2)	10 (35.7)	
Heavy drinker	3 (9.7)	0 (0.0)	
Discharge medication, *n* (%)			
Aspirin	31 (100)	28 (100)	1.000
P2Y_12_ inhibitor			0.026
Clopidogrel	20 (64.5)	9 (32.1)	
Ticagrelor	11 (35.5)	19 (67.9)	
Statin			1.000
Rosuvastatin 20 mg a day	31 (100)	28 (100)	
Beta blocker			0.904
Carvedilol	22 (71.0)	21 (75.0)	
Nebivolol	7 (22.6)	5 (17.9)	
Angiotensin blockade	29 (93.5)	24 (85.7)	0.574
Calcium channel blocker	0 (0.0)	1 (3.6)	0.959
Proton pump inhibitor	28 (90.3)	23 (82.1)	0.592
Loop diuretic	1 (3.2)	2 (7.1)	0.928
Spironolactone	1 (3.2)	0 (0.0)	1.000
Nitrate	3 (9.7)	1 (3.6)	0.680
Trimetazidine	0 (0.0)	2 (7.1)	0.427
Procedural data			
Pre-PCI TIMI flow grade, *n* (%)			0.789
0	24 (77.4)	21 (75.0)	
1	1 (3.2)	2 (7.1)	
2	6 (19.4)	5 (17.9)	
Post-PCI TIMI flow grade, *n* (%)			0.533
2	1 (3.2)	3 (10.7)	
3	30 (96.8)	25 (89.3)	
Multivessel disease, *n* (%)	18 (58.1)	11 (39.3)	0.238
Multivessel PCI, *n* (%)	3 (9.7)	2 (7.1)	1.000
Infarct-related artery, *n* (%)			0.674
Left anterior descending	19 (61.3)	14 (50.0)	
Left circumflex	10 (32.3)	12 (42.9)	
Right coronary	2 (6.5)	2 (7.1)	
Intervention method, *n* (%)			1.000
Drug-eluting stent	29 (93.5)	27 (96.4)	
Bioresorbable scaffold	2 (6.5)	1 (3.6)	
Number of stents	1.1 ± 0.3	1.1 ± 0.3	0.733

BMI, body mass index; COPD, chronic obstructive pulmonary disease; ED, erectile dysfunction; PCI, percutaneous coronary intervention; TIMI, Thrombolysis in Myocardial Infarction.

**Table 5:** Laboratory measurements according to ED classification.

Laboratory measurements	No-to-mild ED	Moderate-to-severe ED	*p* value
	(***n*** = **31)**	**(*****n*** = **28)**	
Baseline lab data: on-admission			
WBC, x10^3^/mm^3^	11.6 ± 3.2	11.8 ± 3.5	0.775
Hb, g/dL	14.9 ± 1.9	15.0 ± 1.2	0.865
Platelet, x10^3^/mm^3^	260.2 ± 60.7	257.7 ± 70.7	0.882
GFR, ml/min/1.73 m^2^ (MDRD)	86.8 ± 18.3	86.6 ± 16.7	0.969
Total cholesterol, mg/dL	203.8 ± 58.0	193.8 ± 41.4	0.452
Triglyceride, mg/dL	235.1 ± 180.5	218.6 ± 148.5	0.705
HDL-C, mg/dL	39.5 ± 11.9	42.5 ± 11.2	0.333
LDL-C, mg/dL	144.8 ± 45.3	128.4 ± 39.7	0.147
VerifyNow, PRU	241.0 ± 42.2	241.7 ± 57.4	0.959
NT-proBNP, pg/ml	711.3 ± 597.1	673.3 ± 666.3	0.824
LV ejection fraction, %	53.7 ± 7.1	53.0 ± 6.0	0.669
Pulse wave velocity, m/sec	1.47 ± 0.27	1.37 ± 0.23	0.149
15.6-7.4,-1.3498ptAnkle-brachial index	1.1 ± 0.1	1.1 ± 0.1	0.556
Follow-up lab data: 6 months			
WBC, x10^3^/mm^3^	7.9 ± 2.3	7.8 ± 2.6	0.965
Hb, g/dL	14.5 ± 1.4	14.5 ± 1.3	0.947
Platelet, x10^3^/mm^3^	234.9 ± 48.1	249.3 ± 70.2	0.368
GFR, ml/min/1.73 m^2^ (MDRD)	91.7 ± 24.5	89.5 ± 19.2	0.710
Total cholesterol, mg/dL	125.2 ± 21.5	127.2 ± 22.5	0.721
Triglyceride, mg/dL	157.4 ± 76.7	163.9 ± 108.8	0.788
HDL-C, mg/dL	42.5 ± 11.8	45.9 ± 13.7	0.303
LDL-C, mg/dL	67.3 ± 18.5	65.4 ± 18.7	0.687
VerifyNow, PRU^a^	113.2 ± 82.5	59.7 ± 76.8	0.013
NT-proBNP, pg/ml	231.2 ± 424.2	143.9 ± 172.7	0.322
LV ejection fraction, %	56.2 ± 6.1	55.3 ± 7.0	0.614
Pulse wave velocity, m/sec	1.36 ± 0.18	1.29 ± 0.16	0.139
Ankle-brachial index	1.2 ± 0.1	1.1 ± 0.1	0.260
EndoPAT	(*n* = 23)	(*n* = 13)	
RHI, %	1.8 ± 0.5	1.8 ± 0.5	0.730

a VerifyNow P2Y12 assay was evaluated at 1-month follow-up, whereas other lab measures were conducted at 6-month follow-up.

Hb, hemoglobin; HDL-C, high-density lipoprotein cholesterol; ED, erectile dysfunction; GFR, glomerular filtration rate; LDL-C, low-density lipoprotein cholesterol; LV, left ventricular; MDRD, Modification of Diet in Renal Disease; PRU, P2Y12 reaction units; RHI, reactive hyperemia index; WBC, white blood cell.

**Table 6:** Questionnaires at 6 months: erectile dysfunction, bleeding and dyspnea according to ED classification.

Questionnaire item	No-to-mild ED	Moderate-to-severe ED	*p* value
	(**n** = **31)**	**(*****n*** = **28)**	
ED category by IIEF-5, *n* (%)			< 0.001
No (22–25)	11 (35.5)	0 (0.0)	
Mild (17–21)	18 (58.1)	0 (0.0)	
Mild to moderate (12–16)	2 (6.5)	0 (0.0)	
Moderate (8–11)	0 (0.0)	12 (42.9)	
Severe (5–7)	0 (0.0)	16 (57.1)	
IIEF-5 score	21.0 ± 3.0	4.8 ± 4.6	< 0.001
Erectile function	4.2 ± 0.7	1.0 ± 1.1	< 0.001
Orgasmic function	4.2 ± 0.7	1.4 ± 1.3	< 0.001
Sexual desire	4.3 ± 0.6	0.9 ± 1.0	< 0.001
Intercourse satisfaction	4.2 ± 0.8	0.8 ± 0.9	< 0.001
Overall satisfaction	4.1 ± 0.7	0.8 ± 1.0	< 0.001
Bleeding (BARC), *n* (%)			0.046
Type 1	13 (41.9)	19 (67.9)	
Type 2	0 (0.0)	1 (3.6)	
Type 3	0 (0.0)	0 (0.0)	
Type 5	0 (0.0)	0 (0.0)	
Dyspnea (mMRC), *n* (%)			0.121
Grade 0	25 (80.6)	18 (64.3)	
Grade 1	6 (19.4)	9 (32.1)	
Grade 2	0 (0.0)	1 (3.6)	
Grade 3	0 (0.0)	0 (0.0)	
Grade 4	0 (0.0)	0 (0.0)	

BARC, Bleeding Academic Research Consortium; ED, erectile dysfunction; IIEF-5, International Index of Erectile Function-5; mMRC, modified Medical Research Council.

### 3.3 . Association between antiplatelet effect and bleeding episode

**Figure 2:**
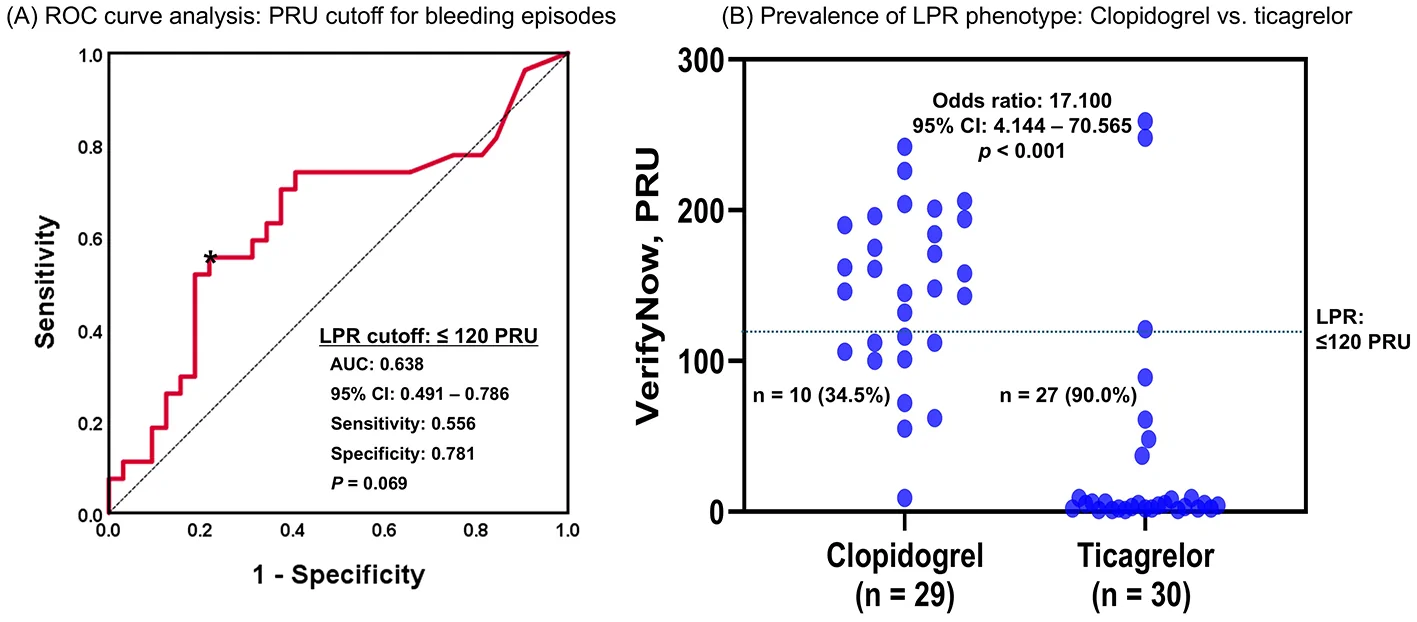
Association between antiplatelet effect and bleeding episodes: **(A)** ROC curve analysis (PRU cutoff for bleeding episodes) and **(B)** prevalence of LPR phenotype (clopidogrel vs. ticagrelor). AUC, area under the curve; CI, confidence interval; LPR, low platelet reactivity; OR, odds ratio; PRU, P2Y12 reaction unit; ROC, receiver operating characteristic.

Platelet reactivity was significantly and inversely associated with the risk of bleeding during the follow-up period (per 10-PRU decrease: OR, 0.926; 95% CI, 0.866–0.990; *p* = 0.024). To assess the discriminatory ability of platelet reactivity for bleeding, we performed ROC curve analysis using PRU values. A PRU cutoff of ≤ 120 was identified as optimal and used to define the low platelet reactivity (LPR) phenotype; its discriminatory performance was modest and did not reach conventional statistical significance (sensitivity 55.6%, specificity 78.1%, AUC 0.638, 95% CI 0.491–0.786, *p* = 0.069) (Figure 2A). However, the LPR phenotype was significantly associated with bleeding risk (67.6% vs. 31.8%: OR, 4.464; 95% CI, 1.441–13.831; *p* = 0.008). The prevalence of the LPR phenotype was significantly higher in the ticagrelor group than in the clopidogrel group (90.0% vs. 34.5%; OR, 17.100; 95% CI, 4.144–70.565; *p* < 0.001) (Figure 2B).

### 3.4 . Factors associated with moderate-to-severe ED

P2Y_12_ inhibitor type, bleeding episodes, and PRU levels differed significantly across ED categories (Tables 4–6). Given the significant association between PRU levels and bleeding episodes, we assessed the combined contribution of P2Y_12_ inhibitor type and bleeding episodes to moderate-to-severe ED. In multivariable analysis, P2Y_12_ inhibitor type and bleeding episodes were jointly associated with the odds of moderate-to-severe ED (Table 7A). Compared with clopidogrel-treated patients without bleeding, ticagrelor-treated patients with bleeding had an approximately 7.2-fold higher odds of moderate-to-severe ED (95% CI, 1.683–30.804; *p* = 0.008). This association remained significant after adjustment for the LPR phenotype (OR, 5.625; 95% CI, 1.052–30.087; *p* = 0.044) (Table 7B), suggesting a potential association beyond ticagrelor's antiplatelet activity.

**Table 7:** Factors associated with moderate-to-severe ED at 6 months post-PCI.

Variable	Odds ratio	95% CI	*p* value
**(A) Model 1: P2Y**_12_ inhibitor type plus bleeding episodes^*^			
Clopidogrel & no bleeding vs.			0.034
Clopidogrel & bleeding	1.200	0.245–5.886	0.822
Ticagrelor & no bleeding	1.600	0.310–8.247	0.574
Ticagrelor & bleeding	7.200	1.683–30.804	0.008
**(B) Model 2: Model 1 plus LPR phenotype** ^*^			
Clopidogrel & no bleeding vs.			0.156
Clopidogrel & bleeding	1.144	0.230–5.696	0.869
Ticagrelor & no bleeding	1.370	0.243–7.723	0.721
Ticagrelor & bleeding	5.625	1.052–30.087	0.044
LPR phenotype ( ≤ 120 PRU)	1.475	0.383–5.682	0.572

CI, confidence interval; ED, erectile dysfunction; LPR, low platelet reactivity; PCI, percutaneous coronary intervention; PRU, P2Y12 reaction unit. ^*^The final models were intentionally kept parsimonious to reduce the risk of overfitting.

## 4 Discussion

**Figure 3:**
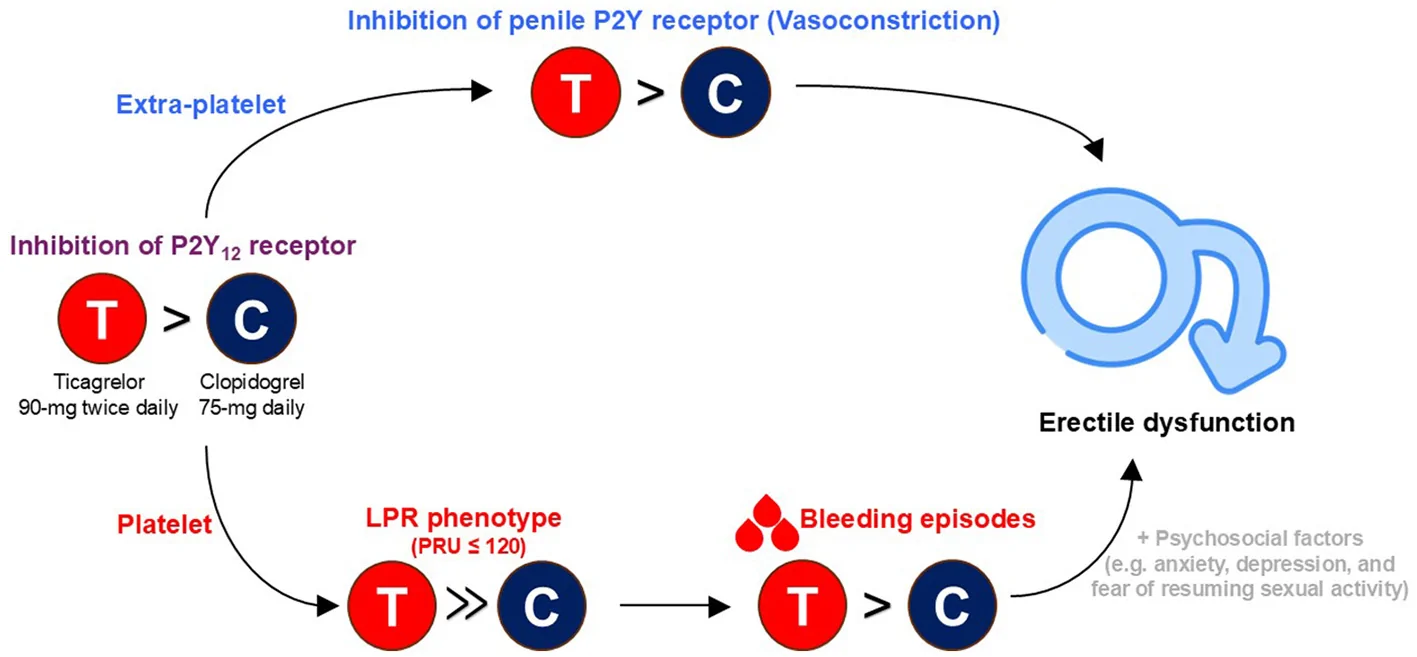
Proposed conceptual framework: association between P2Y_12_ inhibitor type, bleeding episodes, and erectile dysfunction. LPR: low platelet reactivity, PRU: P2Y12 reaction unit.

This study provides novel insights into the association between P2Y_12_ inhibitor type and erectile dysfunction in patients with AMI. Key findings from STEMI patients undergoing primary PCI are summarized as below: (1) ED remained common despite contemporary procedural strategies and GDMT (81.4%); (2) bleeding episodes (predominantly BARC type 1) were frequently observed during 6 months of DAPT (41.4% in clopidogrel and 66.7% in ticagrelor); (3) vascular function measurements (eg, ABI, baPWV, and EndoPAT-RHI) did not differ after 6 months of ticagrelor or clopidogrel therapy; (4) ED severity did not differ according to concomitant CV medications, except for P2Y_12_ inhibitor type; (5) compared with clopidogrel, ticagrelor was associated with approximately 17-fold higher odds of the LPR phenotype, which was in turn associated with more frequent bleeding episodes; and (6) P2Y_12_ inhibitor type (ticagrelor vs. clopidogrel) and bleeding episodes were jointly associated with the odds of moderate-to-severe ED. Compared with clopidogrel, ticagrelor provided more potent P2Y_12_ inhibition and was associated with a higher prevalence of the LPR phenotype and more frequent bleeding episodes. In addition, extraplatelet P2Y_12_ inhibition by ticagrelor in other tissues—potentially including the penile vasculature—may be associated with deterioration in erectile function; however, this remains a hypothesis and is not directly supported by the present data (Figure 3).

The effects of antiplatelet therapy on erectile function remain poorly defined, particularly for P2Y_12_ inhibitors in patients with AMI. Limited prior data suggest a possible association between antiplatelet therapy and sexual dysfunction, although evidence remains inconsistent (12, 13). In the present study, ticagrelor was associated with a significantly higher prevalence of moderate-to-severe ED than clopidogrel, suggesting a potential link between more potent platelet inhibition and impaired erectile function.

The purinergic P2Y_12_ receptor is expressed predominantly on platelets and megakaryocytes, where ADP–P2Y_12_ signaling sustains platelet activation and aggregation. Ticagrelor provides more potent inhibition of the platelet P2Y_12_ receptor than clopidogrel, leading to lower platelet reactivity and a higher incidence of bleeding events—predominantly nuisance bleeding in this study. In AMI patients treated with DAPT, nuisance bleeding is common and independently associated with worse quality of life, including pain, discomfort, dyspnoea, anxiety, and depression (14). Anxiety, depression, fear of recurrent cardiac events, and concerns about resuming sexual activity were not assessed and may represent important unmeasured mediators or confounders. Previous studies have shown that psychological distress after AMI is strongly associated with impaired sexual function and may influence ED more profoundly than nuisance bleeding alone (15). Thus, psychosocial factors—potentially intertwined with bleeding events and treatment awareness—may account for a substantial proportion of the observed association rather than a direct effect of ticagrelor or potent platelet inhibition. Accordingly, although our findings suggest a possible association between ticagrelor treatment and erectile dysfunction, the proposed pathway should be regarded as one of several plausible interpretations rather than a demonstrated biological mechanism.

The mechanism underlying the observed association remains uncertain. Bleeding-related effects on quality of life, unmeasured psychosocial factors, and ticagrelor-specific purinergic or adenosine-mediated effects are plausible pathways; however, none were evaluated in this study, and their relevance to human erectile function remains unknown (4, 5, 16–18). These mechanisms should therefore be considered hypothesis-generating and require validation in prospective mechanistic studies.

After PCI for AMI, DAPT with a potent P2Y_12_ inhibitor (e.g., prasugrel or ticagrelor) is the standard of care for potent platelet inhibition and thrombotic event reduction, especially during the acute phase. During the stable phase, clinical relevance of nuisance bleeding extends well beyond the bleeding event itself. It is among the most frequent complications of DAPT with a potent P2Y_12_ inhibitor and is a major driver of poor treatment adherence (19, 20). In addition, repeated nuisance bleeding may serve as an early clinical signal of an individual patient's heightened bleeding propensity and can prompt reassessment for optimal antiplatelet effect (21). Therefore, growing interest exists in de-escalation strategies during the stable phase to reduce clinically significant bleeding risk without concern for ischaemic events (22, 23). To this end, early aspirin discontinuation and reduced-dose ticagrelor-based regimens (e.g., 60 mg ticagrelor) may be considered in selected patients, but further evidence is needed before these approaches can be adopted as routine strategies (24). Although these findings may encourage greater attention to sexual health during follow-up, they should not be interpreted as supporting changes in antiplatelet therapy solely to improve erectile function. The current study highlights the need to address both CV and quality-of-life domains in patients with STEMI, supporting tailored antiplatelet approaches that incorporate individual patient needs. However, given the observational nature of this substudy, no causal or mechanistic inferences can be drawn from the present findings.

This study has several limitations. First, although treatment allocation was based on the multicenter randomized trial, this erectile dysfunction substudy was conducted at a single participating center and included a relatively small sample. Therefore, selection bias cannot be excluded, and the generalizability of the findings to broader STEMI populations and other clinical settings may be limited. Second, baseline IIEF-5 scores were unavailable because emergent primary PCI precluded pretreatment assessment. Thus, we could not distinguish pre-existing from incident ED or evaluate longitudinal changes, limiting causal inference. Furthermore, unmeasured factors—including prior ED, PDE5 inhibitor use, testosterone status, psychological factors, sexual activity, marital status, and partner availability—may have influenced erectile function independently of antiplatelet therapy. Third, a temporal mismatch existed between the timing of platelet reactivity assessment (at 1-month follow-up) and that of erectile dysfunction (at 6-month follow-up). Nevertheless, platelet reactivity measured at 1 month is likely to reflect the steady-state antiplatelet effect established after PCI in patients with STEMI. Fourth, significant differences in follow-up LDL-C and eGFR levels were observed between treatment groups. The difference in LDL-C levels achieved at 6 months was more likely attributable to differences in baseline LDL-C and individual responses to the same dose of rosuvastatin than to differential medication adherence. Similarly, the lower follow-up eGFR in the ticagrelor group is unlikely to reflect poorer adherence. Fifth, the limited sample size restricted multivariable adjustment and model complexity. Although parsimonious models were used to minimize overfitting, model instability, sparse-data bias, and estimation uncertainty remain possible, as reflected by the wide confidence intervals. The odds ratios should therefore be interpreted primarily as indicating the presence and direction of associations rather than their precise magnitude. Sixth, no mechanistic biomarkers or penile vascular assessments were performed; therefore, the proposed biological mechanisms linking ticagrelor treatment to erectile function remain speculative. Seventh, nearly all bleeding episodes were limited to BARC type 1 nuisance bleeding. Although minor by definition, nuisance bleeding may adversely affect quality of life, undermine confidence in treatment, and contribute to nonadherence or premature antiplatelet discontinuation. However, its cumulative burden, effects on adherence and health status, and subsequent treatment modifications were not systematically assessed. Finally, the analysis focused on erectile function at 6 months and did not assess longer-term outcomes; therefore, whether any observed associations are transient or persistent remains unclear.

## 5 Conclusions

After primary PCI in male patients with STEMI, moderate-to-severe ED and bleeding episodes were common during DAPT. P2Y_12_ inhibitor type (ticagrelor vs. clopidogrel) and bleeding episodes were associated with lower erectile function scores and a higher prevalence of moderate-to-severe ED at 6 months. Given the exploratory nature of this substudy and the lack of baseline erectile function assessment, these findings should be considered hypothesis-generating and warrant confirmation in larger prospective studies.

## Data Availability

The data that support the findings of this study are available from the corresponding author upon reasonable request.

## References

[1] Rao SV O'Donoghue ML Ruel M Rab T Tamis-Holland JE Alexander JH . 2025 ACC/AHA/ACEP/NAEMSP/SCAI Guideline for the Management of Patients With Acute Coronary Syndromes: A Report of the American College of Cardiology/American Heart Association Joint Committee on Clinical Practice Guidelines. Circulation. (2025) 151:e771–862. doi: 10.1161/CIR.0000000000001309 40014670

[2] Vilahur G Gutiérrez M Casani L Lambert C Mendieta G Ben-Aicha S et al. P2Y12 antagonists and cardiac repair post-myocardial infarction: global and regional heart function analysis and molecular assessments in pigs. Cardiovasc Res. (2018) 114:1860–70. doi: 10.1093/cvr/cvy201 30124783

[3] Park Y Koh JS Lee JH Park JH Shin ES Oh JH . HEALING-AMI Investigators. Effect of ticagrelor on left ventricular remodeling in patients with st-segment elevation myocardial infarction (HEALING-AMI). JACC Cardiovasc Interv. (2020) 13:2220–34. doi: 10.1016/j.jcin.2020.08.007 33032710

[4] Patrono C Morais J Baigent C Collet JP Fitzgerald D Halvorsen S . Antiplatelet agents for the treatment and prevention of coronary atherothrombosis. J Am Coll Cardiol. (2017) 70:1760–76. doi: 10.1016/j.jacc.2017.08.037 28958334

[5] Cattaneo M Schulz R Nylander S. Adenosine-mediated effects of ticagrelor: evidence and potential clinical relevance. J Am Coll Cardiol. (2014) 63:2503–9. doi: 10.1016/j.jacc.2014.03.031 24768873

[6] Dehmer GJ Badhwar V Bermudez EA Cleveland JC Jr Cohen MG D'Agostino RS . 2020 AHA/ACC Key data elements and definitions for coronary revascularization: a report of the american college of cardiology/american heart association task force on clinical data standards (writing committee to develop clinical data standards for coronary revascularization). Circ Cardiovasc Qual Outcomes. (2020) 13:e000059. doi: 10.1161/HCQ.0000000000000059 32202924

[7] Salonia A Bettocchi C Boeri L Capogrosso P Carvalho J Cilesiz NC . EAU working group on male sexual and reproductive health. european association of urology guidelines on sexual and reproductive health-2021 update: male sexual dysfunction. Eur Urol. (2021) 80:333–57. doi: 10.1016/j.eururo.2021.06.007 34183196

[8] Mehran R Rao SV Bhatt DL Gibson CM Caixeta A Eikelboom J . Standardized bleeding definitions for cardiovascular clinical trials: a consensus report from the Bleeding Academic Research Consortium. Circulation. (2011) 123:2736–47. doi: 10.1161/CIRCULATIONAHA.110.009449 21670242

[9] Jeong YH Oh JH Yoon HJ Park Y Suh J Lee SW . Pharmacodynamic profile and prevalence of bleeding episode in east asian patients with acute coronary syndromes treated with prasugrel standard-dose versus de-escalation strategy: a randomized A-MATCH trial. Thromb Haemost. (2021) 121:1376–86. doi: 10.1055/a-1346-3300 33401330

[10] Mahler DA Wells CK. Evaluation of clinical methods for rating dyspnea. Chest. (1988) 93:580–6. doi: 10.1378/chest.93.3.580 3342669

[11] ParkHW KangMG AhnJH BaeJS TantryUS GurbelPA . Effects of monotherapy with clopidogrel vs. aspirin on vascular function and hemostatic measurements in patients with coronary artery disease: the prospective, crossover I-LOVE-MONO. Trial J Clin Med. (2021) 10:2720. doi: 10.3390/jcm1012272034202960 PMC8235752

[12] IrfanM IsmailSB NoorNM HussainNHN. Efficacy of aspirin for vasculogenic erectile dysfunction in men: a meta-analysis of randomized control trials. Am J Mens Health. (2020) 14:1557988320969082. doi: 10.1177/155798832096908233111628 PMC7607788

[13] LiR PengL DengD LiG WuS. Potential causal association between aspirin use and erectile dysfunction in European population: a Mendelian randomization study. Front Endocrinol. (2024) 14:1329847. doi: 10.3389/fendo.2023.132984738260164 PMC10800513

[14] AminAP BachuwarA ReidKJ ChhatriwallaAK SalisburyAC YehRW . Nuisance bleeding with prolonged dual antiplatelet therapy after acute myocardial infarction and its impact on health status. J Am Coll Cardiol. (2013) 61:2130–8. doi: 10.1016/j.jacc.2013.02.04423541975 PMC4332538

[15] SmithAB DavisM JacksonEA WittmannD SmithJ BartonDL. Sexual function, anxiety, depression and coping after myocardial infarction: an exploratory study. Sex Disabil. (2022) 40:77–89. doi: 10.1007/s11195-021-09715-x36712231 PMC9881546

[16] OdomMR PakES BrownDA HannanJL. Enhanced electrical field stimulated nitrergic and purinergic vasoreactivity in distal vs proximal internal pudendal arteries. J Sex Med. (2017) 14:1285–96. doi: 10.1016/j.jsxm.2017.09.01329110801 PMC5828175

[17] GurS SikkaSC KnightGE BurnstockG HellstromWJ. Purinergic contraction of the rat vas deferens in L-NAME-induced hypertension: effect of sildenafil. Asian J Androl. (2010) 12:415–21. doi: 10.1038/aja.2009.7020305675 PMC3739254

[18] Faria M Magalhães-Cardoso T. Lafuente-de-Carvalho JM, Correia-de-Sá P. Corpus cavernosum from men with vasculogenic impotence is partially resistant to adenosine relaxation due to endothelial A(2B) receptor dysfunction. J Pharmacol Exp Ther. (2006) 319:405–13. doi: 10.1124/jpet.106.107821 16837560

[19] Ben-Dor I Torguson R Scheinowitz M Li Y Delhaye C Wakabayashi K . Incidence, correlates, and clinical impact of nuisance bleeding after antiplatelet therapy for patients with drug-eluting stents. Am Heart J. (2010) 159:871–5. doi: 10.1016/j.ahj.2010.01.016 20435198

[20] Kwon TJ Tantry US Park Y Choi YM Ahn JH Kim KH . Influence of platelet reactivity on BARC classification in East Asian patients undergoing percutaneous coronary intervention. Results of the ACCEL-BLEED study. Thromb Haemost. (2016) 115:979–92. doi: 10.1160/TH15-05-0366 26790469

[21] BiscagliaS TonetE PavasiniR SerenelliM BuganiG CimagliaP . A counseling program on nuisance bleeding improves quality of life in patients on dual antiplatelet therapy: a randomized controlled trial. PLoS ONE. (2017) 12:e0182124. doi: 10.1371/journal.pone.018212428832589 PMC5568410

[22] Gorog DA Ferreiro JL Ahrens I Ako J Geisler T Halvorsen S . De-escalation or abbreviation of dual antiplatelet therapy in acute coronary syndromes and percutaneous coronary intervention: a Consensus Statement from an international expert panel on coronary thrombosis. Nat Rev Cardiol. (2023) 20:830–44. doi: 10.1038/s41569-023-00901-2 37474795

[23] KangMG AhnJH KimK KohJS ParkJR HwangSJ . Prevalence of adverse events during ticagrelor versus clopidogrel treatment and its association with premature discontinuation of dual antiplatelet therapy in East Asian patients with acute coronary syndrome. Front Cardiovasc Med. (2022) 9:1053867. doi: 10.3389/fcvm.2022.105386736578832 PMC9791044

[24] Kim MH Jeong YH. Protocol update and issues in EASTYLE trial. J Cardiovasc Intervention. (2024) 3:89–97. doi: 10.54912/jci.2024.0004

