# Supplementary material for: Impact of antiplatelet therapy on erectile dysfunction following percutaneous coronary intervention in patients with ST-segment elevation myocardial infarction
Source: Front Med (Lausanne). 2026 Sep 3;13:1874442. doi: 10.3389/fmed.2026.1874442 (PMC13581904; doi:10.3389/fmed.2026.1874442)
Supplement: Supplementary file 1 [file Table_1.docx]

**Supplementary Table 1. The contents of the questionnaires survey at 6-month follow-up in STEMI patients**

**1. Erectile dysfunction: international index of erectile function-5 (IIEF-5) scoring**

| **Over the past 6 months:** | | | | | |
| --- | --- | --- | --- | --- | --- |
| **Score** | **1** | **2** | **3** | **4** | **5** |
| **1. How do you rate your confidence that you could get an erection?** | Very low | Low | Moderate | High | Very high |
| **2. When you had erections with sexual stimulation, how often were your erections hard enough for penetration?** | Almost never/ Never | A few times | Sometimes | Most times | Almost always/ Always |
| **3. During sexual intercourse, how often were you able to maintain your erection after you had penetrated (entered) your partner?** | Almost never/ Never | A few times (much less than half the time) | Sometimes (about half the time) | Most times (much more than half the time) | Almost always/ Always |
| **4. During sexual intercourse, how difficult was it to maintain your erection to completion of intercourse?** | Extremely difficult | Very difficult | Difficult | Slightly difficult | Not difficult |
| **5. When you attempted sexual intercourse, how often was it satisfactory for you?** | Almost never/ Never | A few times (much less than half the time) | Sometimes (about half the time) | Most times (much more than half the time) | Almost always/ Always |
| **IIEF-5 scoring: The IIEF-5 score is the sum of the ordinal responses to the 5 items.**  22-25: No erectile dysfunction  17-21: Mild erectile dysfunction  12-16: Mild to moderate erectile dysfunction  8-11: Moderate erectile dysfunction  5-7: Severe erectile dysfunction | | | | | |

**2. Bleeding: Bleeding Academic Research Consortium (BARC) criteria.**

| **Type** | **Definition** |
| --- | --- |
| **0** | No evidence of bleeding |
| **1** | Bleeding that is not actionable |
| **2** | Any clinically over sign of hemorrhage that is actionable, but does not meet criteria for type 3, 4, or 5 bleeding. It must meet at least one of the following criteria:  - Requiring medical or percutaneous intervention guided by a   healthcare professional  - Leading to hospitalization or an increased level of care  - Prompting evaluation (laboratory or imaging) |
| **3** | Clinical, laboratory, and/or imaging evidence of bleeding with healthcare responses, as listed below: |
| **3a** | - Any transfusion with overt bleeding - Overt bleeding plus hemoglobin drop ≥3 to <5 g/dl (provided Hb   drop is related to bleeding) |
| **3b** | - Overt bleeding plus Hb drop ≥5 g/dl - Cardiac tamponade - Bleeding requiring surgical intervention for control - Bleeding requiring intravenous vasoactive drugs |
| **3c** | - Intracranial hemorrhage - Intraocular bleed compromising vision |
| **4** | CABG-related bleeding - Perioperative intracranial bleeding within 48 hours - Reoperation following closure of sternotomy for the purpose of   controlling bleeding - Transfusion of ≥5 units of whole blood or packed red blood cells   within a 48-hour period - Chest tube output ≥2L within a 24-hour period |
| **5** | Fatal bleeding. Bleeding directly causes death with no other explainable cause. Categorized further as either definite or probable: |
| **5a** | Probable fatal bleeding that is clinically suspicious as the cause of death, but the bleeding is not directly observed |
| **5b** | Definite fatal bleeding that is directly observed |

**3. Dyspnea: Modified Medical Research Council (mMRC) scale**

| **Grade 0** | I only get breathless with strenuous exercise |
| --- | --- |
| **Grade 1** | I get short of breath when hurrying on the level or walking up a slight hill |
| **Grade 2** | On level ground, I walk slower than people of the same age on the level because of breathlessness, or I have to stop for breath when walking at my own pace |
| **Grade 3** | I stop for breath after walking about 100 yards or after a few minutes on level ground |
| **Grade 4** | I am too breathless to leave the house, or I am breathless when dressing |
